# Viral-Cellular DNA Junctions as Molecular Markers for Assessing Intra-Tumor Heterogeneity in Cervical Cancer and for the Detection of Circulating Tumor DNA

**DOI:** 10.3390/ijms18102032

**Published:** 2017-09-22

**Authors:** Katrin Carow, Mandy Gölitz, Maria Wolf, Norman Häfner, Lars Jansen, Heike Hoyer, Elisabeth Schwarz, Ingo B. Runnebaum, Matthias Dürst

**Affiliations:** 1 Department of Gynecology, Jena University Hospital—Friedrich Schiller University Jena, 07747 Jena, Germany; katrin.carow@med.uni-jena.de (K.C.); mandy.goelitz@med.uni-jena.de (M.G.); mariawolf1990@gmx.de (M.W.); norman.haefner@med.uni-jena.de (N.H.); lars.jansen@med.uni-jena.de (L.J.); ingo.runnebaum@med.uni-jena.de (I.B.R.); 2 Institute of Medical Statistics, Information Sciences and Documentation, Jena University Hospital—Friedrich Schiller University Jena, 07743 Jena, Germany; heike.hoyer@med.uni-jena.de; 3 Research Program Infection and Cancer, DKFZ, 69120 Heidelberg, Germany; e.schwarz@dkfz-heidelberg.de

**Keywords:** HPV, viral-cellular junction, molecular marker, tumor heterogeneity, cell-free tumor DNA

## Abstract

The development of cervical cancer is frequently accompanied by the integration of human papillomaviruses (HPV) DNA into the host genome. Viral-cellular junction sequences, which arise in consequence, are highly tumor specific. By using these fragments as markers for tumor cell origin, we examined cervical cancer clonality in the context of intra-tumor heterogeneity. Moreover, we assessed the potential of these fragments as molecular tumor markers and analyzed their suitability for the detection of circulating tumor DNA in sera of cervical cancer patients. For intra-tumor heterogeneity analyses tumors of 8 patients with up to 5 integration sites per tumor were included. Tumor islands were micro-dissected from cryosections of several tissue blocks representing different regions of the tumor. Each micro-dissected tumor area served as template for a single junction-specific PCR. For the detection of circulating tumor-DNA (ctDNA) junction-specific PCR-assays were applied to sera of 21 patients. Samples were collected preoperatively and during the course of disease. In 7 of 8 tumors the integration site(s) were shown to be homogenously distributed throughout different tumor regions. Only one tumor displayed intra-tumor heterogeneity. In 5 of 21 analyzed preoperative serum samples we specifically detected junction fragments. Junction-based detection of ctDNA was significantly associated with reduced recurrence-free survival. Our study provides evidence that HPV-DNA integration is as an early step in cervical carcinogenesis. Clonality with respect to HPV integration opens new perspectives for the application of viral-cellular junction sites as molecular biomarkers in a clinical setting such as disease monitoring.

## 1. Introduction

Genetic diversity is not only characteristic among tumors of the same entity but also within the tumor itself. The latter is often referred to as intra-tumor heterogeneity and reflects the co-existence of different tumor sub-clones. Indicators for intra-tumor heterogeneity are somatic copy number alterations [1], point mutations [2,3], differences in ploidy [4] or different transcript expression patterns [5]. The phenomenon needs to be considered when using individualized biomarkers in a clinical setting, for example during postoperative monitoring of cancer patients based on the detection of circulating tumor-derived DNA in serum or plasma. The potential value of this strategy has been demonstrated for a variety of cancer types [6,7,8,9], but so far, the impact of intra-tumor heterogeneity on biomarker performance has not been assessed systematically. Navin and colleagues report the existence of totally independent sub-clones in breast tumors [4]. Due to Darwinian selection, these sub-clones could grow into local relapse or distant metastases that differ in their genetic make-up from the primary tumor. In the absence of a common genetic aberration it would be necessary to perform multiple biopsies to identify a biomarker specific for each sub-clone in order to reliably detect persistent or recurrent disease based on liquid biopsies. This illustrates clearly the importance of a profound knowledge of the tumor’s genomic landscape and mutations common to all cells for optimal biomarker design.

The clonal composition of cervical cancer has been addressed in several studies. A frequently applied technique for assessing clonal relation of cancer cells in women is the analysis of the X-chromosome inactivation pattern [10]. Using this strategy, Hu and colleagues have analyzed 24 preparations of the same cervical carcinoma and revealed its polyclonal origin [11]. Similar studies report polyclonality in 2 of 8 cases [12] as well as a monoclonal history of 6 examined adenosquamous cervical cancers [13]. However, since there is a 50% probability that two independent subpopulations carry the same inactivated X-chromosome, additional biomarkers are required to rule out heterogeneity. Another informative approach suitable for the investigation of intra-tumor heterogeneity is comparative genomic hybridization. Lyng and colleagues could demonstrate heterogeneity in chromosomal aberrations in 11 of 20 analyzed advanced cervical cancers [14]. However, a polyclonal history was ruled out due to the fact that the majority of the detected aberrations were present in all analyzed tumor regions.

Evidence is cumulating that human papillomaviruses (HPV) DNA integration may be particularly interesting in evaluating intra-tumor heterogeneity. A prerequisite for cervical cancer development is a persisting infection with an oncogenic type of HPV [15,16]. During carcinogenesis, HPV DNA is frequently integrated into the host genome. This is discussed as being an early event [17]. Integration is significantly associated with lesion progression [18,19], and evidently contributes to clonal expansion [17]. Integration frequencies in cervical carcinomas up to 81.7% have been reported [20]. Early studies used the physical state of the HPV genome in different tumor regions as a marker for clonality and provide evidence for monoclonal [13] as well as polyclonal lesions [21,22]. However, these studies were based on 2D-gel electrophoresis or HPV genome fragment ratios, which only provide indirect evidence for virus integration. In this context, the detection of HPV DNA integrates on sequence level is a far more specific and innovative approach: The uniqueness of each integration event renders the viral-cellular junctions as perfect molecular markers for tracing tumor cells. A first proof of feasibility was provided by Vinokurova and colleagues who used junction sequences as clonal markers to demonstrate a common history of vulvar and previous cervical lesions in 4 of 5 patients [23]. Should viral-cellular-junction sequences turnout to be representative of the whole tumor cell population, their detection may become a powerful tool in patient monitoring. In this context, Campitelli and colleagues provided a first prove of strategy value and demonstrated that viral-cellular junction sequences can be used to detect circulating tumor-derived DNA (ctDNA) in sera from 11 of 13 patients [24]. By analyzing sequential serum samples, they could correlate their findings with tumor recurrence.

However, thus far the distribution of viral-cellular junction sequences throughout the tumor mass has not been analyzed. Prior to a clinical implementation of HPV-integrates as biomarkers for disease monitoring it is essential to clarify the issue of intra-tumor heterogeneity. Our study addresses this topic systematically by analyzing DNA from micro-dissected tissue of tumors by amplification of the viral-cellular junction (vcj-PCR). Tumors with single and multiple integration sites were included. To assess the potential value of viral cellular junction sequences for disease monitoring, we performed vcj-PCRs with cell-free DNA (cfDNA) from sera of cervical cancer patients to identify circulating tumor DNA (ctDNA). The data were analyzed with respect to clinical parameters and patient outcome.

## 2. Results

To test our hypothesis that viral-cellular junction sequences are suited to serve as highly specific tumor markers for cervical carcinomas, we first addressed cervical cancer clonality by assessing the distribution of viral-cellular junction sequences in eight cervical cancers. We aimed to evaluate the influence of intra-tumor heterogeneity on biomarker performance. In the second part, we tested the suitability of viral-cellular junction sequences to serve as molecular markers for the detection of circulating tumor DNA by analyzing cell-free DNA from serum samples by vcj-PCR. Overall, we included tissue and serum samples of 21 patients with integrated HPV DNA and established (semi)-nested vcj-PCR-assays for 32 viral-cellular junctions (Table 1).

### 2.1. The Majority of Tumors Show Intra-Tumor Homogeneity with Respect to Junction Distribution

The specimens available for assessing intra-tumor heterogeneity comprised tumor tissue which was dissected into 3–11 (I-XI) individual blocks of up to 0.5 cm^3^ in size. On basis of HE- and p16-staining of corresponding sections, tumor and stroma islands were identified and micro-dissected by laser capture technology for PCR analyses. For tumors with multiple junctions, areas in close proximity to each other or the corresponding areas of a subsequent section were selected for micro-dissection.

**Figure 1:**
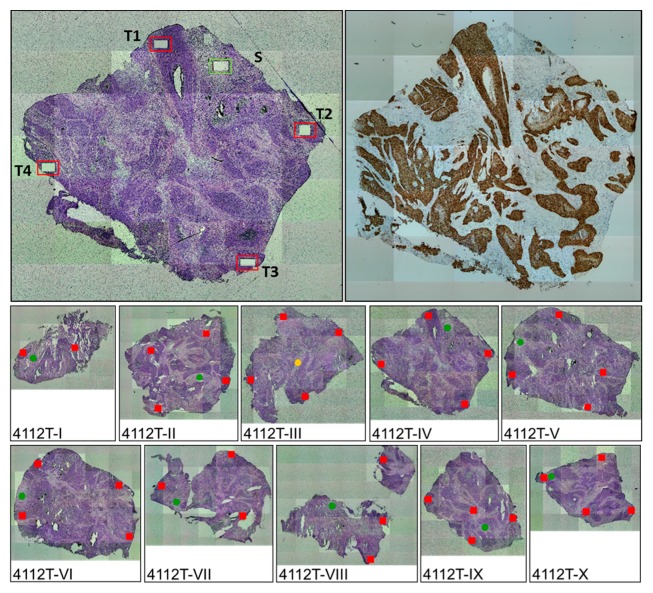
Intra-tumor heterogeneity analyses. Ten tissue blocks (I-X) of tumor 4112 were available. Left upper panel: Hematoxylin and eosin (HE)-stained section of block IV after micro-dissection; S: stroma, T1 to T4: tumor. (**Right upper**) panel: p16-staining of block IV to guide micro-dissection; (**Middle and lower**) panel: HE stained sections of all blocks. Squares and circles refer to micro-dissected tumor and stroma areas, respectively. Red, and green colors indicate the presence and absence of the viral junction, respectively. Yellow indicates a marginally positive result.

Tumors harboring single HPV integrates (*n* = 4) invariably tested positive for the respective junction sequence in all analyzed areas (Figure 1).

**Figure 2:**
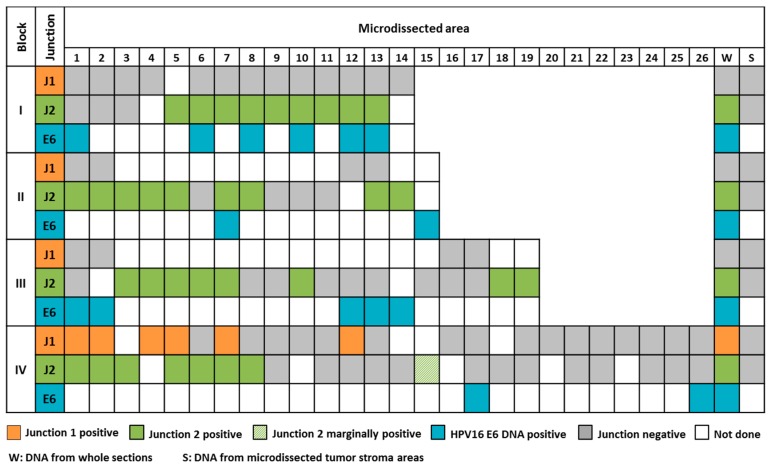
Varying numbers of areas were micro-dissected from four blocks of tumor 4977 and submitted to junction-specific PCR. Areas tested for the presence of both junctions received identical numbers. Successful detection is indicated by color. Both junctions are heterogeneously detected throughout the four blocks. Junction 1 is present in block IV only whereas junction 2 is detectable in all blocks, but not in all areas.

Likewise, 3 of 4 carcinomas harboring between 2 and 5 integrates showed a homogenous distribution of the viral integrates (Figure S1). Irregular junction distribution was observed in tumor 4977 only. We analyzed 4 blocks of this tumor and identified numerous regions in all blocks which displayed exclusively junction 2. Only 6 of 22 analyzed areas harbored junction 1, in four of them in coexistence with junction 2. Moreover, multiple areas were negative for both junction fragments (Figure 2 and Figure S2). vcj-PCRs performed with DNA from whole tissue sections of all blocks showed no evidence for presence of junction 1, except for block IV. For a subset of junction-negative tumor areas we additionally tested for the presence of HPV E6 DNA and obtained a positive result indicative for viral episomes or further unidentified integrates. All stroma areas (*n* = 8) were invariable negative for the junction sites. Thus, tumor 4977 clearly constitutes a case of intra-tumor heterogeneity with respect to junction distribution.

### 2.2. Detection of Viral-Cellular Junctions Fragments in Cell-Free Serum DNA

After demonstrating that viral-cellular junction sequences are in most cases representative for the respective tumor, we analyzed the suitability of the fragments to serve as marker for the detection of circulating tumor DNA. We tested pre-operative sera of all 8 patients included in the intra-tumor heterogeneity analyses as well as pre-operative sera of 13 additional cervical cancer patients (Table 2). Circulating cell-free DNA was isolated from 200 µL serum resulting in 50 µL of purified serum DNA.

Junction detection by (semi-) nested PCR was performed in triplicates with 4 µL template each and verified by Southern Blot hybridization using an internal oligonucleotide as probe. Cell-free serum-DNA of five of 21 patients was tested positive, providing clear evidence for the presence of tumor DNA (Table 2). Among cases displaying multiple integrates, junction detection was only successful in serum of patient 5189. Notably, all 3 junction fragments could be amplified. The serum corresponding to tumor 4977, which displayed intra-tumor heterogeneity, was negative for both known junctions.

**Figure 3:**
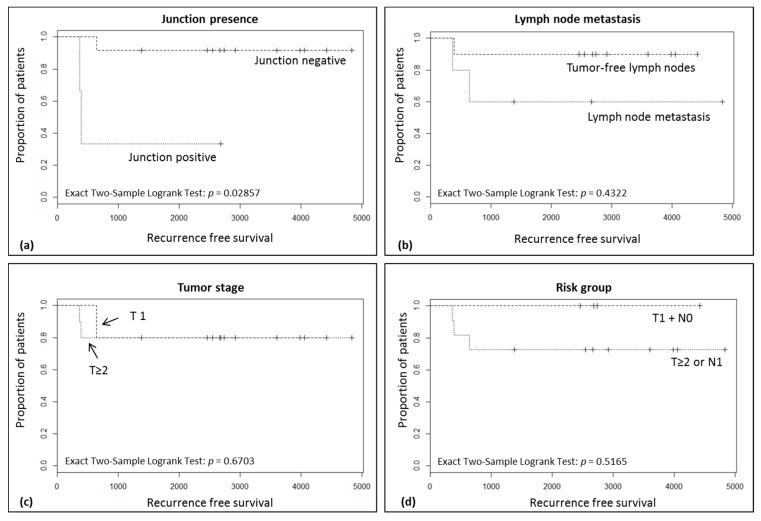
Kaplan Meier curves to assess the impact of (**a**) junction presence, (**b**) lymph node metastasis, (**c**) tumor stage and (**d**) the combined presence of risk factors on recurrence free survival. Censored subjects are indicated with a tick mark: (+).

No correlation was found between the detection of viral-cellular junctions and TNM-stage (Fisher’s exact test). However, Kaplan Meier analyses of the patients with primary tumors only revealed a significant association between the detection of junction fragments in pre-operative sera and a reduced recurrence free survival (*p* = 0.03) (Figure 3a). In the same cohort there was no correlation between reduced recurrence free survival and established risk factors like lymph node metastasis (*p* = 0.43), an advanced tumor stage (*p* = 0.67) or a combination thereof (*p* = 0.52) (Figure 3b–d).

Of 9 patients sequential serum samples were available which were collected during second surgery or at one of the follow up visits (Table S1). Samples of all 3 patients whose primary serum samples displayed junction fragments remained positive. Two of these patients had second surgery within 82 days. The third positive case was patient 4338 with one serum sample taken during surgery of the primary tumor and a second one taken 1.5 years later during relapse surgery. Both sera displayed junction presence. In follow up serum samples of the 6 initially negative patients no junctions could be detected despite one case of therapy failure (patient 1907).

## 3. Discussion

Viral-cellular junction sequences frequently arise during cervical carcinogenesis as a result of HPV DNA integration into the host genome. These integration sites are unique and highly specific for a patient’s tumor and may thus serve as versatile tumor makers. Ideally, a tumor marker is representative for the entire tumor cell population. Intra-tumor heterogeneity typically observed in the course of clonal expansion complicates tumor biomarker research immensely. To determine the potential value of HPV DNA integration as a tumor biomarker, it is essential to assess whether HPV integrates are stable over time and space, i.e., whether they are representative for the tumor cell population. To address this, we analyzed 8 cervical cancers with regard to intra-tumor heterogeneity concerning the distribution of viral-cellular junction sequences. In this context, all 4 cervical carcinomas harboring single HPV integrates showed homogeneity. In case of tumors with multiple integrates, we found that 3 of 4 tumors harbored all tumor specific integrates in every micro-dissected tissue area in each tumor block, thereby implying monoclonal origin. Tumor 841 is of particular interest as it harbors 5 integrates on 3 different chromosomes (Table 1). On two chromosomes the integrates are in proximity of each other, possibly as a result of local rearrangements [25]. Since all 5 integrates were detected in all micro-dissected areas at least 3 independent integration events and two chromosomal rearrangements must have taken place in one common precursor cell. Both forms of integrates are also evident for tumor 5189 which harbors 3 integrates on 2 chromosomes. In contrast, tumor 1509 exclusively displays 2 integrates on 2 different chromosomes, which are likewise distributed homogenously. This extensive homogeneity in integrate distribution provides striking evidence for the importance of integration in an early phase of carcinogenesis and for its presumptive driving effect during clonal expansion. Moreover, it would appear that integrates that may have resulted from chromosomal rearrangements occurred at the same point in time. The only exception is tumor 4977. This tumor exhibits 2 integrates on different chromosomes and was the only case of irregular junction distribution. Junction 1 was highly underrepresented and detected in only 14% (6/43) of the tested areas. Junction 2 was present in 54% (34/63) of the tested areas. A co-detection of both junctions was evident in 4 tumor areas (Figure 2 and Figure S2). Areas without detectable junctions, either harbor unidentified integrates, viral episomes only or both. The evident irregular integration pattern in this tumor and its relevance for carcinogenesis is open for all kinds of speculations including gain or loss of integrates at a later stage of tumor development.

Evidently, HPV DNA integration can be an early event in cervical carcinogenesis. In 7 of 8 examined tumors HPV integration has most likely driven clonal expansion. The homogeneous integrate distribution is also a strong indication that the continuous presence of the integrated viral DNA in the tumor is required for the maintenance of the carcinogenic phenotype. These observations, in particular the presence of specific integration site(s) representative for all tumor cells, highlight their usefulness as molecular markers for disease monitoring. 

This extensive homogeneity of the driver event is highly remarkable in the light of the ambiguous situation of driver genetic event distribution in other tumor entities: human epidermal growth factor receptor 2 *HER2* gene amplification is an accepted driver event in breast cancer development. However, this aberration is not always detectable throughout the whole tumor tissue [26,27]. In contrast, Gerlinger and colleagues analyzed ten clear cell renal carcinomas and report that chromosome 3p loss and von Hippel–Lindau gene (*VHL*) aberrations were the only ubiquitous events, whereas 73–75% of identified driver aberrations were of subclonal origin [28]. In non-small cell lung cancer (NSCLC), de Briun et al. report that the majority of driver events seem to be clonal, although there are cases of heterogeneity [29]. On the other hand, a study by Zhang et al. describe heterogeneity in *TP53* mutations status in *TP53* driven NSCLC [30]. Compared to the complex genomic landscape of other tumor entities the unique virus induced etiology of cervical cancer allows a facilitated and precise analysis of clonal architecture. Our results clearly demonstrate a high selective pressure for integrate maintenance and the suitability of viral-cellular junction sequences for biomarker research. 

Limitations of our study include the small number of patients and the use of micro-dissected tissue areas rather than single cells. However, clear draw-backs of single cell analyses are false negative results, especially if only one copy of the target molecule is present. The micro-dissected tissue areas used in our study represent 100 to 200 cells, which allow reliable analyses. In our study, intra-tumor homogeneity implies that the tumor-specific integrate(s) were detected in all micro-dissected areas taken within a tumor comprising up to several cm^2^. To be scored positive the ∆*C*_t_ values had to correspond to a tumor cell fraction of ≥25% within a dilution series (Figure S3). Moreover, our approach does not allow drawing general conclusions about intra-tumor heterogeneity in cervical cancer as it focusses on viral integration sites only. Clearly, as in any other tumor entity, subclones evolve with distinct genetic alterations such as chromosomal aberration, DNA copy number changes and gene mutations.

To explore the use of integration sites as molecular markers for disease monitoring or prognosis we developed patient specific vcj-PCRs for the detection of circulating tumor DNA (ctDNA) in serum. We tested pre-operative sera of all 8 individuals included in above intra-tumor heterogeneity analyses as well as pre-operative sera of 13 additional cervical cancer patients (Table 1 and Table 2). For 9 of 21 patients post-operative samples were also available (Table S1). Junction fragments were detectable in pre-operative 5 of five of 21 patients (23%). A similar study by Campitelli and colleagues demonstrated a detection rate of 68% [24]. Our low frequency of junction presence in sera may be explained by the fact that only 3 replicates corresponding to 48 µL serum were analyzed. In the former study 10 replicates corresponding to 160 µL serum were analyzed, with less than 50% of replicates being positive in the majority of cases. These stochastic effects seen in both studies clearly indicate that the analyses of some sera were done at the detection limit. Only 4 of the 7 sera of patients with proven intra-tumor homogeneity displayed junction fragments. Of note is that all 3 junctions characteristic for tumor 5189 could be detected in the corresponding serum. This co-detection of multiple viral-cellular junction sequences in serum has not been demonstrated before and implies that each junction is equally suited to serve as tumor marker. Not surprisingly, neither of the 2 junction fragments was detected in the serum of patient with tumor 4977 which showed intra-tumor heterogeneity. For more comprehensive results DNA from larger serum volumes need to be analyzed and quantified with respect to junction presence. This can be achieved by digital PCR [31].

Among the cases of relapse, we found a higher, albeit non-significant, detection rate of junction fragments in comparison to the sera of patients with primary tumors (60% vs. 21%). Also Campitelli and colleagues hypothesize that ctDNA is more easily released from a relapse than from a primary tumor. The detection of a relapse during monitoring would benefit from such a phenomenon.

Another novel finding of this study is that among the group of patients with primary tumors the detection rate of junction fragments in serum correlated significantly with a reduced recurrence-free survival (*p* = 0.03) (Figure 3a). A prognostic value of ctDNA presence before therapy has also been reported for other tumor entities such as colorectal [32,33], metastatic breast cancer [6] or metastatic uveal melanoma [34]. A further limitation of our study is the restricted number of patients, the different group size of junction positive and junction negative patients. As a consequence, the absence of a prognostic value of known established risk factors (Figure 3b–d) could be related to the limited number of subjects.

Finally, we evaluated the suitability of vcj-PCR based ctDNA detection to follow tumor dynamics. We analyzed 15 sequential serum samples of 9 patients. Three patients had junction fragments in pre-operative serum collected at primary surgery. Two of these patients required second surgery within 82 days at which a second serum sample was collected. Not surprisingly, the junction fragments were also detectable in the respective second serum sample. For the 6 patients without junction fragments in the sera taken at primary surgery, none of the follow up sera tested positive (Table S1). These patients were also free from tumor recurrence. Of particular interest is patient 4338 who relapsed close to two years after primary tumor resection. The junction fragment identified in the primary tumor was detectable in both sera of the patient illustrating the stability of the virus integration site over time.

In conclusion, we were able to use the unique viral etiology of cervical cancer to address tumor clonality in the context of intra-tumor heterogeneity and to open new perspectives for biomarker design. 

## 4. Materials and Methods

### 4.1. Samples and Sample Processing

Tissue and serum samples of this retrospective study were obtained from a total of 21 cervical carcinoma patients undergoing treatment at Jena University Hospital between 1996 and 2006. Approval was given by the Ethics Committee of the Friedrich-Schiller University Jena (reference numbers 0175-02/00 and 2174-12/07, 20 September 2017). Written informed consent was obtained from all participants. All patients received an ID, which constitutes the number under which the first sample was archived. Further samples from the same patient were continuously numbered. Each tumor had been analyzed in previous studies for presence and site of HPV integrates [35,36] Tumors were HPV 16 (*n* = 13) or HPV 18 (*n* = 8) positive, of squamous cell (*n* = 18) or of glandular (*n* = 3) origin and ranged between FIGO (Fédération Internationale de Gynécologie et d’Obstétrique) stages IB and IVA (Table 1). Large tumors were cut into smaller blocks. For heterogeneity analyses, 3–11 blocks of each tumor representing different tumor areas were available. Frozen sections of these blocks were used for micro-dissection and DNA isolation.

Tumor cell content of sections was assessed after p16 and Ki67 immuno-staining. p16 overexpression and Ki67 are characteristic for HPV-transformed cells and cell proliferation, respectively. Cryosections of 7 µm thickness were stained on glass slides (Thermo Fisher Scientific, Waltham, MA, USA) with hematoxylin and eosin using standard protocols. For immunohistochemistry 7 µm cryosections (Thermo Fisher Scientific, Waltham, MA, USA) were fixed on Superfrost Plus slides with 4% paraformaldehyde for 10 min. Slides were washed with tris-buffered saline (50 mM Tris, 150 mM NaCl) and 0.1% Tween-20 (TBST) and incubated in 0.6% H_2_O_2_ for 7 min. After another washing step, blocking was performed with goat serum (1:5 dilution in TBST) for 20 min. Staining was performed over night with a Ki67 mouse monoclonal antibody clone mib-1 (DAKO, 80 mg/L), diluted at a ratio of 1:100 or with a p16 mouse monoclonal antibody clone E6H4 ready for use (CINtec Histology, Roche, Basel, Switzerland). For detection, the DAKO EnVision System was used according to the manufacturer’s protocol. Finally, slides were counterstained with hematoxylin and covered with coverslips in gelatin.

Tumor sections for micro-dissection were stained with kresyl violet to enable tumor and stroma island identification while minimizing DNA damage. For this purpose, 15 µm-sections were placed on membrane slides (MembraneSlides NF, PALM Microlaser Technologies, Zeiss, Jena, Germany) and dried at room temperature. Staining was performed with 1% kresyl violet for 2 min. Afterwards, samples were washed twice with 70% ethanol and dried at room temperature.

DNA isolated from whole sections was used for evaluating assay sensitivity and as positive control. Ten consecutive 10 µm cryosections of each tissue specimens were digested with proteinase K overnight. DNA was isolated with QIAamp DNA Mini Kit (Qiagen, Hilden, Germany) following manufacturer’s instructions.

Serum samples were used for ctDNA analyses. Blood was collected pre-operatively at the time point of surgical resection of primary tumor (*n* = 17) or of relapse (*n* = 4). From 9 patients, additional samples were available, taken pre-operatively at further surgeries or during follow up (Table 1 and Table S1). For coagulation the blood was stored for 30 min at room temperature. Afterwards it was centrifuged at 3000 rpm for 10 min and serum was stored at −30 °C. For isolation of cell-free DNA, 200 µL aliquots of sera were processed with the High Pure Viral Nucleic Acid Kit (Roche Applied Science, Mannheim, Germany) according to manufacturer’s instructions. The 50 µL-eluat was purified by NaCl-EtOH-precipitation. The pellet was dissolved in 50 µL aqua dest. Quality and quantity of serum-DNA was controlled in β-Actin qPCR.

### 4.2. Intra-Tumor Heterogeneity Analyses: Assay Design and Optimization

For the detection of HPV-integrates by vcj-PCR, primers were designed to flank the junction of human and viral DNA. For heterogeneity analyses, a duplex nested PCR was performed (Table S2). In the first reaction the 3′junction and the housekeeping gene beta globin (HBB) were amplified in parallel in a standard Eppendorf PCR cycler. The first reaction step (50 °C, 2 min, for UDG-reaction) was followed by initial denaturation at 95 °C for 10 min. After 15 cycles (95 °C, 15 s denaturation; 20 s 58–63 °C annealing; 30 s, 72 °C elongation) and 2 min at 72 °C for, 1 µL of the reaction was transferred to two separate reactions, respectively, for amplification of the two templates with internal primer pairs. The second PCR was performed with 40 cycles using the same cycling conditions but in a real-time format (Rotor Gene Q 5plex HRM, Qiagen, Hilden, Germany). Finally, product identity was confirmed by melting curve analyses. The first duplex reaction mixture consisted of 10 ng template (sensitivity tests) or of one micro-dissected tissue area (heterogeneity analyses), 10 µL 2× Platinum^®^ Quantitative PCR SuperMix-UDG (Thermo Fisher Scientific, Waltham, MA, USA), 0.25 µM external primers for vcj-PCR, 0.006–0.25 µM primers for HBB-amplification and was adjusted to 20 µL with aqua dest. Micro-dissected tissues were directly lysed in the entire PCR reaction mixture. The second simplex PCR consisted of 5 µL Fast Start Universal SYBRGreen-Master Mix (Roche Applied Science, Mannheim, Germany), 0.25 µM of each internal primer, 1 µL template and was adjusted to 10 µL with aqua dest.

Sensitivity and specificity of vcj-primers was tested in serial dilutions of DNA isolated from tumor sections in a background of DNA derived in equal parts from HeLa and Caski cells. The combination of both cell lines was chosen to illustrate the absence of any cross reactivity with unspecific HPV integrates. Each dilution step contained 5 ng/µL DNA with decreasing amounts of tumor-DNA (100%, 50%, 25%, 5%, 1%, 0.2%, 0.02%, 0.002% and 0%). The resulting *C*_t_-values of the second PCR enabled a semi-quantitative analysis by calculating Δ*C*_t_ values between HBB- and vcj-PCR Ct. Based on the dilution series Δ*C*_t_ values were calculated for each assay to score the micro-dissected tissue as “clearly positive” (tumor cell fraction of ≥25%), “marginally positive” (tumor cell fraction of 0.02–5%) and “negative” for virus integrate presence (Figure S3).

### 4.3. Intra-Tumor Heterogeneity Analyses: Laser Micro-Dissection

Micro-dissection was performed with the help of the PALM laser capture microscope (Zeiss, Jena, Germany) and the PALM-ROBO-Software. For heterogeneity analyses tumor and stroma areas (20.000 μm^2^) equivalent to 100–200 cells were dissected. Stroma served as control for specificity. In case of multiple integrates, areas in close proximity to each other or identical areas in consecutive sections were selected. Each dissected tissue area was transferred with a sterile needle to a reaction tube, which was directly supplemented with the complete PCR mix containing all necessary reagents. The PCR was performed as described above without further DNA-isolation. The results were evaluated according to Δ*C*_t_-values.

### 4.4. ctDNA Analyses: Detection of Viral-Cellular Junctions in Cell-Free Tumor DNA from Sera

For ctDNA-detection, (semi-) nested-PCR primers were designed to flank all 33 junctions of 21 patients (Table S3). In contrast to DNA deriving from tissue, cell-free serum DNA is highly fragmented. Consequently ctDNA-pimers were designed to minimize product size. Performance of junction-specific PCR (vcj-PCR) assays was optimized in serial dilutions of tumor tissue-DNA in a background of HPV-negative genomic DNA from C33A cells. All reactions were performed in a 20 µL volume using the Fast Start Universal SYBR Green PCR Mastermix (Roche Applied Science, Mannheim, Germany) with 0.25 µM of forward and reverse primer, respectively. After initial denaturation (95 °C, 10 min) 15 amplification cycles (95 °C, 15 s; 55–66 °C, 40 s) were performed in a standard Eppendorf cycler. From this first reaction 2 µL were transferred to the second PCR comprising (semi-) nested primers. Amplification was done in 45 cycles under identical conditions as in the first PCR, but in a real-time format (Rotor Gene Q 5plex HRM, Qiagen, Hilden, Germany) and was followed by a dissociation stage for product identification.

Optimized vcj-primers were used for serum analyses with 4 µL serum-DNA as template. The (semi-) nested PCR approach was performed as described above. The results were verified by Southern Blot with an internal oligonucleotide as hybridization probe (Table S4). For this purpose, PCR products were separated by agarose gelelectrophoresis, denatured by treatment with blot buffer (0.6 N NaCl/0.4 N NaOH) for 3 × 20 min and transferred onto a nylon membrane. Membrane was washed with 2× SSC (sodium chloride sodium citrate for 2 × 10 min and crosslinked using a GS Gene Linker (Bio-Rad, Hercules, CA, USA) Prehybridization was done for two hours at 37 °C in 6× SSC, 5× Denhardt’s, 0.02 M NaPP, 0.5% SDS and 100 µg/mL yeast total RNA. Hybridization was done in 6× SSC, 1× Denhardt’s, 0.02 M NaPP, 100 µg/mL yeast total RNA and y^32^-ATP labeled internal oligonucleotide. Labelling reaction was performed with Polynucleotide Kinase (Roche Applied Science, Mannheim, Germany) following manufacturer’s instructions with 6 pmol of internal oligonucleotide. Using mini Quick Spin Columns (Roche Applied Science, Mannheim, Germany) product was purified following manufacturer’s instructions. Hybridization was done overnight. Hybridization temperature was calculated from internal oligonucleotide melting temperature: T(Hybridisation) = T_M_ (Oligo) − 7 °C. Afterwards, the membrane was washed in 6× SSC and 0.02 M NaPP for 3 × 10 min at 37 °C and for 2 × 30 min at T_M_ (Oligo) − 5 °C. Exposition was done with Kodak X-OMAT radiographic films.

### 4.5. Statistical Analyses

The association between junction-detection and Tumor, Node, Metastasis-stage was analyzed by Fisher exact test. Survival analyses were performed with the statistical software R (package “coin”). Significance was evaluated by log-rank exact test with a two-sided significance level of 0.05.

## Figures and Tables

**Table 1:** Patient material.

Patient-ID	Age at Diagnosis	Histologic Subtype	Tumor Type	HPV Type	TNM-Classification			HPV-Integrates	3′ Integration Site	Tumor Blocks for ITH Analyses	Serum Samples for ctDNA Analyses
					T	N	M				
841	58	SCC	P	16	2a	0	1	1	2q33.3	6	3
								2	13q22.2		
								3	13q22.1		
								4	Xp22.11		
								5	Xp22.11		
1509	70	SCC	P	16	2b	1	0	1	9p13.3	6	1
								2	8q23.3		
1907	33	SCC	P	16	4			1	14q23.2	-	3
2349	58	SCC	P	16	1b1	0	0	1	Xp22.31	-	4
2555	48	SCC	P	16	2a	1	0	1	1p36.22	3	2
2723	52	SCC	P	16	2b	0	x	1	17q23.1	-	3
3817	42	SCC	P	16	1b1	1	0	1	16q23.1	-	1
3986/4112 *	47	SCC	P	18	3b	1		1	17p13.1	10	2
4154	46	SCC	P	18	1b	0	x	1	2q24.2	-	1
4338	60	SCC	P	16	2	0	x	1	9p24.1	11	1
4497	49	ADC	P	18	2b	0	x	1	3p24.2	-	2
4502	37	ADC	P	18	3b	1	x	1	7q34	-	1
4749	40	SCC	P	16	1b1	0	0	1	18p11.32	-	1
								2	18p11.32		
								3	18q12.2		
4977	67	SCC	P	16	2b	0	0	1	6q22.32	4	1
								2	7p15.1		
5234	33	SCC	P	16	1b2	0		1	3p21.31	5	2
5254	29	SCC	P	18	2b2	0		1	9p21.3	-	1
5613	37	ADC	P	18	1b1			1	8p12	-	1
3719	38	SCC	R	16	1b	1	0	1	2p22.3	-	1
								2	4q21.1		
4601	63	SCC	R	16	4	0	x	1	9p23	-	1
4995	52	SCC	R	18	2b	0	0	1	19p13.3	-	1
5189	48	SCC	R	16	1b2	0	0	1	8q24.21	3	1
								2	8q23.2		
								3	5p14.1		

HPV: human papillomaviruses; ITH: intra-tumor heterogeneity; ADC: adenocarcinoma; SCC: squamous cell carcinoma; P: primary tumor; R: relapse; TNM-Classification: Tumor, Node, Metastasis; * patient received two primary tumor surgeries yielding samples 3986 and 4112, sample 4112 served for heterogeneity analyses.

**Table 2:** Junction detection in primary serum samples and follow-up of patients.

Tissue	Patient	Follow-Up (Days)	Status at End of Follow-Up	Junctions	Junction-Detection in Primary Serum	Junction-Detection in Sequential Sera
**Primary Tumor**	841	2549	deceased	J1	No	No
				J2	No	No
				J3	No	No
				J4	No	No
				J5	No	No
	1509	2663	deceased	J1	No	x
				J2	No	x
	1907	257	Deceased *	J1	No	No
	2349	2462	tumor-free	J1	No	No
	2555	4831	tumor-free	J1	No	No
	2723	3605	tumor-free	J1	No	No
	3817	643	Deceased *	J1	No	x
	3986	360	Deceased *	J1	Yes	Yes
	4154	4420	tumor-free	J1	No	x
	4338	1350	Deceased *	J1	Yes	Yes
	4497	4055	tumor-free	J1	No	No
	4502	1376	deceased	J1	No	x
	4749	0	tumor-free	J1	No	x
				J2	No	x
				J3	No	x
	4977	3980	tumor-free	J1	No	x
				J2	No	x
	5234	2681	tumor-free	J1	Yes	Yes
	5254	2923	tumor-free	J1	No	x
	5613	2740	tumor-free	J1	No	x
**Relapse**	3719	-	Deceased *	J1	No	x
				J2	No	x
	4601	-	Deceased *	J1	Yes	x
	4995	-	Deceased *	J1	No	x
	5189	-	Deceased *	J1	Yes	x
				J2	Yes	x
				J3	Yes	x

* tumor-related death; x: not done.

## Supplementary Materials

Supplementary materials can be found at www.mdpi.com/1422-0067/18/10/2032/s1.

## Abbreviations

ADC	Adenocarcinoma
cfDNA	Circulating cell-free DNA
Ct	Cycle threshold
ctDNA	Circulating tumor DNA
FIGO	Fédération internationale de gynécologie et d’obstétrique
HE	Hematoxylin and eosin stain
HPV	Human papillomavirus
ITH	Intra-tumor heterogeneity
NSCLC	Non-small cell lung cancer
SCC	Squamous cell carcinoma
T_M_	Melting temperature
vcj-PCR	Viral-cellular junction PCR
