# Supplementary material for: Viral-Cellular DNA Junctions as Molecular Markers for Assessing Intra-Tumor Heterogeneity in Cervical Cancer and for the Detection of Circulating Tumor DNA
Source: Int J Mol Sci. 2017 Sep 22;18(10):2032. doi: 10.3390/ijms18102032 (PMC5666714; doi:10.3390/ijms18102032)
Supplement: Supplementary file 1 [file ijms-18-02032-s001.pdf]

Supplementary Materials: Viral-cellular DNA junctions as molecular markers for assessing intra-tumor heterogeneity in cervical cancer and for the detection of circulating tumor DNA

Katrin Carow, Mandy Gölitz, Maria Wolf, Norman Häfner, Lars Jansen, Heike Hoyer, Elisabeth Schwarz, Ingo B. Runnebaum and Matthias Dürst

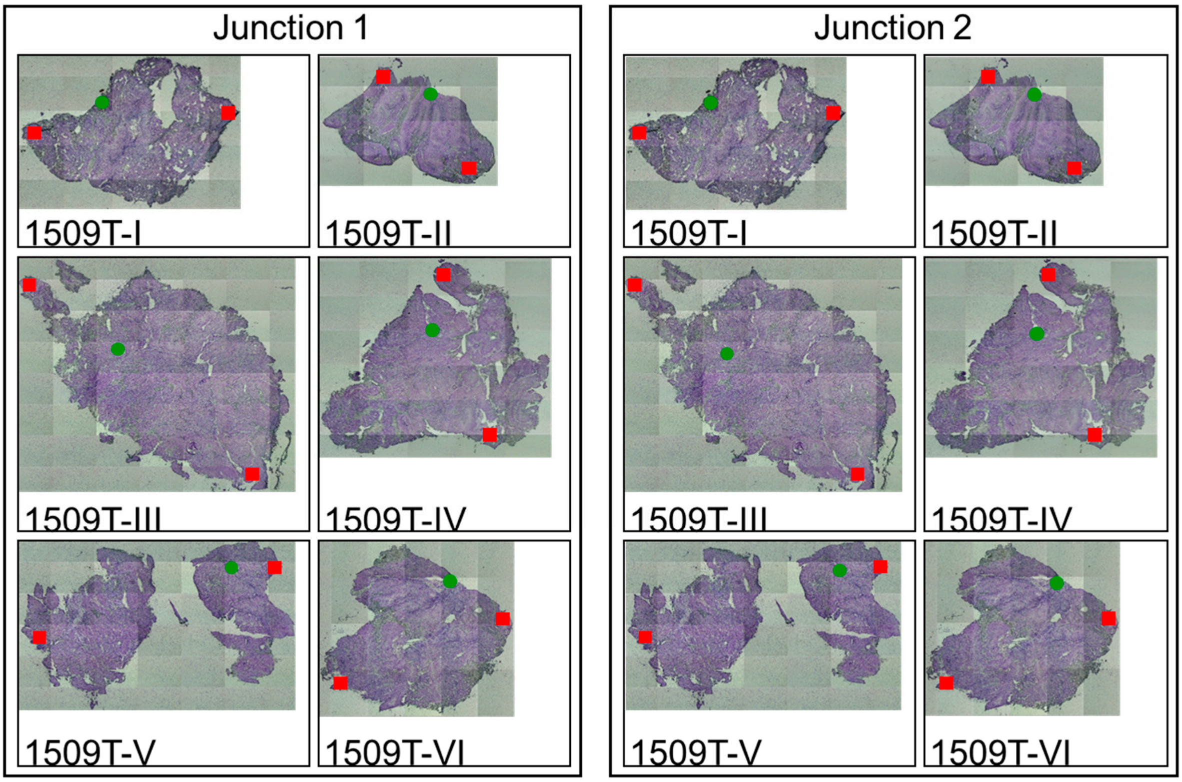

Figure S1. Intra-tumor heterogeneity analyses. Six tissue blocks (I-VI) of tumor 1509 were available. Left panel: Detection of junction 1; right panel: Detection of junction 2. Squares and circles refer to tumor and stroma areas, respectively. Red, and green colors indicate the presence and absence of the viral junction, respectively.

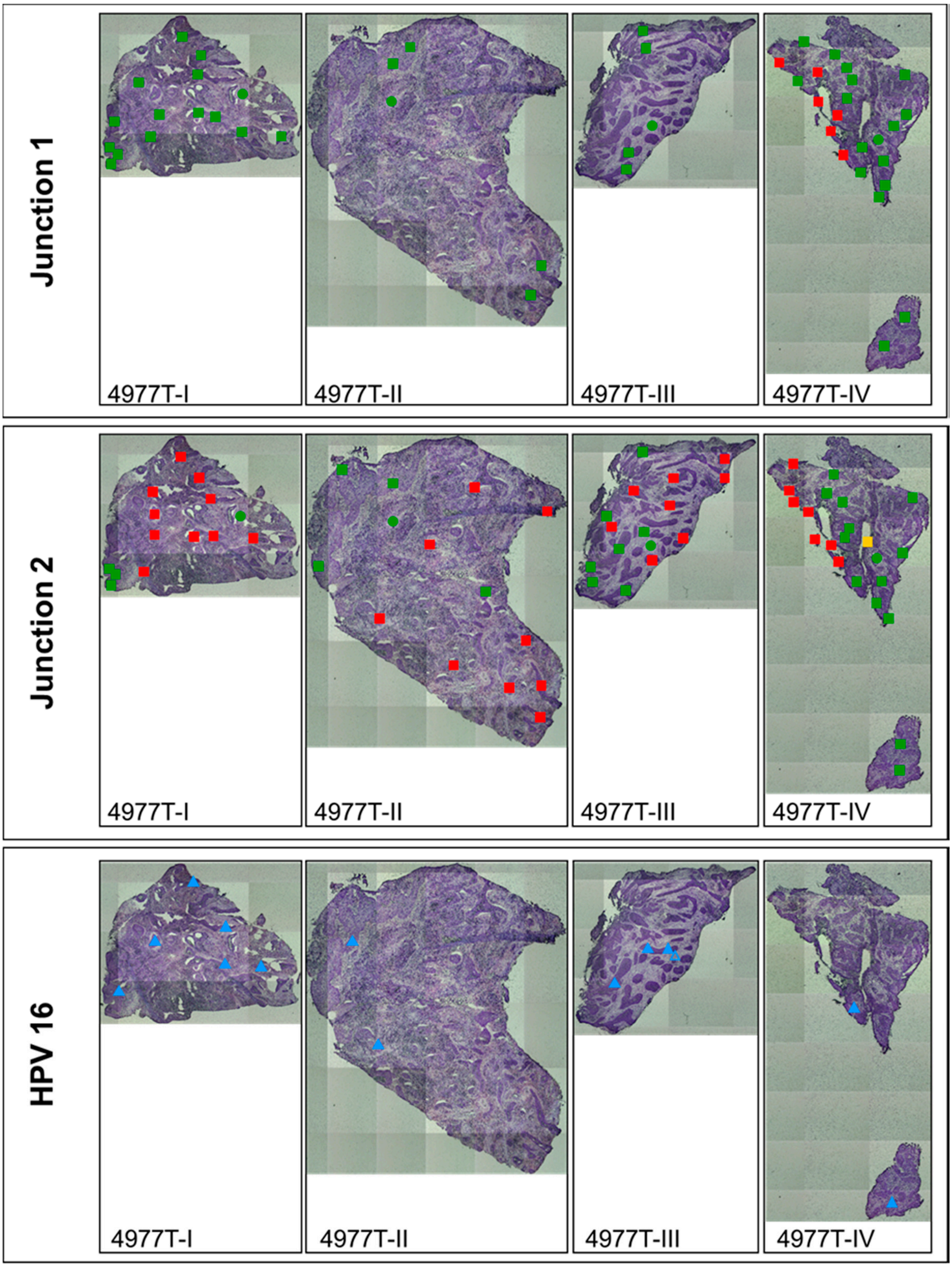

Figure S2. Intra-tumor heterogeneity analyses. Four tissue blocks (I-IV) of tumor 4977 were available. Upper panel: Detection of junction 1; middle panel: Detection of junction 2. Squares and circles refer to tumor and stroma areas, respectively. Red, and green colors indicate the presence and absence of the viral junction, respectively. Yellow indicates a marginally positive result. Lower panel: Blue triangles indicate the presence of HPV16 DNA.

		delta ct								
	tumor cell fraction	100%	50%	25%	5%	1%	0,2%	0,02%	0,002%	0%
tumor DNA		10ng	5ng	2,5ng	0,5ng	0,1ng	0,02ng	0,002ng	0,0002ng	0ng
tumor cells		2000	1000	500	100	20	4	0,4	0	0
**T2319**	20-30	-1,44	1,80	1,79	4,21	6,36	8,55	10,18		
**T5240**	75-85	-0,57	0,39	1,51	4,28	7,60	9,04			
**T4112**	40-50	1,62	1,43	3,23	5,42	8,47	13,23			
**T4338**	50,00	-2,36	-1,13	0,10	2,66	5,51	7,56	10,25		
**T841 J1**	35-45	-5,22	-3,92	-1,80	1,03	3,81	6,99	11,27		
**T841 J2**	35-45	-2,33	-1,18	-0,01	2,91	4,79	6,62			
**T841 J3**	35-45	-2,60	-1,26	0,30	3,08	5,15				
**T841 J4**	35-45	-1,36	0,59	1,14	3,23	5,35	8,88			
**T841 J5**	35-45	-5,64	-4,65	-3,89	-1,64	0,95	3,39	6,44		
**T4977 J1**	50-60	-0,22	0,97	2,00	5,77	8,27				
**T4977 J2**	50-60	-0,23	1,66	4,05	7,82					
**T5189 J1**	<20	-0,53	2,59	4,23	6,83	9,62	10,29			
**T5189 J2**	<20	6,99	11,83	13,86	19,02	18,72				
**T5189 J3**	<20	-0,78	1,88	4,23	7,30	9,22	9,72			
**T1509 J1**	60-70	-4,40	-2,52	-2,18	0,76	2,84	5,00			
**T1509 J2**	60-70	3,76	6,71	8,86	11,84	15,83				

Figure S3. Intra-tumor heterogeneity analyses. Dilution series of tumor DNA extracted from whole tissue sections in a background of non-tumor DNA in order to determine the cut-off levels for the detection of viral-cell junctions in micro-dissected tissue. The resulting Ct-values (HBB- and vcj-PCR) of the second PCR enabled a semi-quantitative analysis for junction presence. ΔCt values in red, yellow and green indicate a "clearly positive", "marginally positive" and "negative" result, respectively for the presence of the junction fragment.

Table S1. Serum samples for longitudinal analyses

Patient	Junction-Detection in Primary Sample	Sequential Sample	Time from 1st Sample [days]	Reason for Taking	Tumor-Free?	Junction Detection
841	No	**1159**	161	Follow-up	Yes	No
		**3502**	1064	Follow-up	Yes	No
1907	No	**1965**	21	2nd surgery	No	No
		**2066**	79	Follow-up	No	No
2349	No	**2523**	85	Follow-up	Yes	No
		**2964**	274	Follow-up	Yes	No
		**3397**	463	Follow-up	Yes	No
2555	No	**4589**	1251	Follow-up	Yes	No
2723	No	**3424**	298	Follow-up	Yes	No
		**4565**	833	Follow-up	Yes	No
3986	Yes	**4112**	82	2nd surgery	No	**Yes**
4338	Yes	**4978**	705	Relapse surgery	No	**Yes**
4497	No	**4687**	202	Follow-up	Yes	No
5234	Yes	**5240**	6	2nd surgery	No	**Yes**

Table S2. Viral-cellular junction primer for intra-tumor heterogeneity analyses

Junction	vcj-PCR Primer (5′→3′) for Intra-Tumor Heterogeneity Analyses							
	External PCR		TA (°C)	bp	Internal PCR		TA (°C)	bp
841, J1	**F**	TGGTGAAACCCCGTCTTTAC	61	365	**F**	TGGTGAAACCCCGTCTTTAC	61	195
	**R**	TGATGGAGGTGATTGGAAGC			**R**	TGAAATTTCTGCAAGGGTCTG		
841, J2	**F**	GTGGAAGTGCAGTTTGATGG	61	324	**F**	AACTGTGGTAGAGGGTCAAGTT	61	186
	**R**	CCCACTGTTGAGCCTGAGTAG			**R**	CCCACTGTTGAGCCTGAGTAG		
841, J3	**F**	TATGGAGACACGCCAGAATG	61	204	**F**	CACGCCAGAATGGATACAAA	61	196
	**R**	GTGGGGCTGGGATTTTATCT			**R**	GTGGGGCTGGGATTTTATCT		
841, J4	**F**	GATATCCCAGGGTGCATCAC	61	289	**F**	CCGGAGGAGAGGATTTGAGT	61	168
	**R**	CATCTGTGTTTAGCAGCAACG			**R**	CATCTGTGTTTAGCAGCAACG		
841, J5	**F**	CGATTGGTGTATTGCTGCAT	58	228	**F**	CATGGGGAATGGTTGTGTTA	58	121
	**R**	GGCCAGGACACTGTTACTTTG			**R**	GGCCAGGACACTGTTACTTTG		
1509, J1	**F**	TGCACAGGAAGCAAAACAAC	63	258	**F**	TGCACAGGAAGCAAAACAAC	61	199
	**R**	GTGCAGTGGTGTGATCTTGG			**R**	CCACCTCAGCGTCCCTAGTA		
1509, J2	**F**	ACGAAAACGGAAATCCAGTG	63	293	**F**	ACGAAAACGGAAATCCAGTG	61	280
	**R**	TGGACAAGTCCCATTGTCAG			**R**	TTGTCAGCCTGAACCCTACA		
2555/ 2319	**F**	ACAACTCCCAGCCTGACAAG	58	243	**F**	GTGAAGTCACGGGTAGGG	58	130
	**R**	AATAAACTGTTATCACTTAACAATGC			**R**	CTTAACAATGCGACACAAACG		
3986/ 4112	**F**	GACTGAGACTCCATCTCAAAACAA	58	200	**F**	TGAATTTTTACCTGCCAGTGC	58	105
	**R**	TCAGATGACGAGGACGAAAA			**R**	AGACACAGGGTCGGATATGG		
4338	**F**	TGATAGACCTACATGTGCCAAACTT	61	185	**F**	TGATAGACCTACATGTGCCAAACTT	58	114
	**R**	TGACGAGAACGAAAATGACAGTGA			**R**	TAACACAGGCAGAAACAGAGACG		
4977, J1	**F**	TGGGAGTTTCCCTACACACG	63	381	**F**	TGGGAGTTTCCCTACACACG	61	300
	**R**	TATGGAGACACGCCAGAATG			**R**	ATGGTACAATGGGCCTACGA		
4977, J2	**F**	AAACACATGCGCCTAGAATG	63	419	**F**	AAACACATGCGCCTAGAATG	61	247
	**R**	TCCCCACACCCTTCTGTTAC			**R**	TGGTGCGTTAAACACTCTCG		
5189, J1	**F**	TGATGGAGGTGATTGGAAGC	63	309	**F**	TGATGGAGGTGATTGGAAGC	61	260
	**R**	CCACCATGCCCTCTAATTTC			**R**	CATGTTAGCCAGGATGGTC		
5189, J2	**F**	CATTAGCAGATGCCAAAATAGG	63	463	**F**	CATTAGCAGATGCCAAAATAGG	61	350
	**R**	AGGTCCTTCACATCCATTGC			**R**	TAAGAATGCCTTCACATCCA		
5189, J3	**F**	GTGGAAGTGCAGTTTGATGG	63	355	**F**	GTGGAAGTGCAGTTTGATGG	61	215
	**R**	CAAGGGTTATTTGGATGTTGTG			**R**	GCAAGAAACACCTTGTCCTCA		
5240	**F**	GCTGAGGCAGGAGAATTGC	58	188	**F**	GAGGCAGAGGTTGTAGTGAGC	58	126
	**R**	CATAGATGACAATTTAAGAAATGC			**R**	AAATTTAGTTTCTATGGATGTAAAGC		

Table S2. *Cont.*

Target	Further PCR Primer (5′→3′) for Intra-Tumor Heterogeneity Analyses							
	External PCR		T_A (°C)	bp	Internal PCR		T_A (°C)	bp
HBB	F	AGTCAGGGCAGAGCCATCTA	58-61	158	F	ACACAACTGTGTTCACTAGC	58-63	110
	R	CCTCACCACCAACTTCATCC			R	CAACTTCATCCACGTTCACC		
HPV16 E6	F	AATGTTTCAGGACCCACAGG	60	338	F	CAGGACACACAGTGGCTTTTGA	60	204
	R	CAGGACACACAGTGGCTTTTGA			R	TTGCTTTTCGGGATTTATGC		

Table S3. Primer for ctDNA analyses

Junction	vcj-PCR Primer (5′→3′) for ctDNA Analyses							
	External PCR		T_A (°C)	bp	Internal PCR		T_A (°C)	bp
841, J1	F	TGGTGAAACCCCGTCTTTAC	55	195	F	TGGTGAAACCCCGTCTTTAC	58	146
	R	TGAAATTTCTGCAAGGGTCTG			R	TTTTTGGTTACAACCATTAGCAGA		
841, J2	F	AACTGTGGTAGAGGGTCAAGTT	58	238	F	GGAATACGAACATATTTTGTGCAG	58	186
	R	CCCACTGTTGAGCCTGAGTAG			R	CCCACTGTTGAGCCTGAGTAG		
841, J3	F	TATGGAGACACGCCAGAATG	61	204	F	TATGGAGACACGCCAGAATG	61	121
	R	CCCCTCCATGGTAAAGAATG			R	ACACGTTTTCCGAAAGCAAA		
841, J4	F	CCGGAGGAGAGGATTTGAGT	58	168	F	CCGGAGGAGAGGATTTGAGT	58	134
	R	CCGGAGGAGAGGATTTGAGT			R	TGAAATTATTAGGCAGCACTTGG		
841, J5	F	TGCGATTGGTGTATTGCTG	61	198	F	GAACATGTTGTCTGTCTGGTCA	63	165
	R	ATGGTACAATGGGCCTACGA			R	TGCGATTGGTGTATTGCTG		
1509, J1	F	TGCACAGGAAGCAAAACAAC	61	258	F	TGCACAGGAAGCAAAACAAC	55	199
	R	GTGCAGTGGTGTGATCTTGG			R	CCACCTCAGCGTCCCTAGTA		
1509, J2	F	ACGAAAACGGAAATCCAGTG	61	204	F	GTATCAGGATTTAGTATTACTAC	61	133
	R	GATGTATGTGAGTGATTGGCGTA			R	GATGTATGTGAGTGATTGGCGTA		
1907	F	CAGCCCTCCTTTGTTCTCC	66	241	F	CAGCCCTCCTTTGTTCTCC	58	187
	R	TGGTACAATGGGCCTACGAT			R	TATGCACAATTGGCAGACAC		
2349	F	ACATGCGCCTAGAATGTGCT	63	205	F	ACATGCGCCTAGAATGTGCT	66	185
	R	AAGAAGCTGGGGTGATGATG			R	TCCAAGAACAGGAGAAGTGTCT		
2555	F	ACACCCATGGCTGAATCTCT	63	159	F	GTGAAGTCACGGGTAGGG	58	130
	R	CTTAACAATGCGACACAAACG			R	CTTAACAATGCGACACAAACG		
2723	F	GTGCTTCCCTCGAGTTTCC	63	195	F	CATTTCAGGTTATTGTTTACTCATCC	58	125
	R	GCAGCACTATATTGGTATAGAACAGG			R	GGGAGACACACCTGAGTGG		
3719, J1	F	CTCAAGGACGTGGTCCAGAT	61	219	F	ACGAGGACAAGGAAAACGA	55	184
	R	TAGCATTACCGGGCTTCAG			R	TAGCATTACCGGGCTTCAG		

3719, J2	F	GGTACAACTAAAATGCCCTCCA	61	210	F	GGTACAACTAAAATGCCCTCCA	55	161
	R	CCTCCCTCCTTGGTCATCTAC			R	CCTCCCTCCTTGGTCATCTAC		
3817	F	CTTTGTTGCCCAGGCTAGAG	55	224	F	GTGCAATCTTGGTTCACTGC	61	130
	R	GCCATGAGACTGAAACACCA			R	GGAAGTGGGGGTGGTTGC		
3986	F	GACTGAGACTCCATCTCAAAACAA	63	200	F	TGAATTTTTACCTGCCAGTGC	63	105
	R	TCAGATGACGAGGACGAAAA			R	AGACACAGGGTCGGATATGG		
4154	F	ATGTCCCATGCAATATTTGG	55	238	F	ATCTGGCCGCCTATCTACAA	58	150
	R	AAGGGGGAGATTGGAGACC			R	AAGGGGGAGATTGGAGACC		
4338	F	GAAACAAAAAGAAGAAACCCAGAG	61	162	F	TGATAGACCTACATGTGCCAAACTT	58	114
	R	TAACACAGGCAGAAACAGAGACG			R	TAACACAGGCAGAAACAGAGACG		
4497	F	CACCAGTCACACAGACAAGGAAC	61	191	F	CACCAGTCACACAGACAAGGAAC	58	160
	R	CACAAATATACATCCAGCAAAGGA			R	TGGCCATATTTAGAAAGTAGAATAACA		
4502	F	CTGGGAGATGGACGTTGC	58	196	F	GATCGTGCCACTGAACTCC	55	97
	R	TCCAGCAAAGGATAATAGATGG			R	CATTTGATAAAAATGGCAATCC		
4601	F	AGGTACCAATGGGGAAGAGG	58	169	F	AGGTACCAATGGGGAAGAGG	61	148
	R	TTCAAGAGGTCTGGGAGGTG			R	TGATGAATTGATTTTAACAAGTGTG		
4749, J1	F	AAGAGGGTACGGGATGTAATGG	63	198	F	TGACGAGAACGAAAATGACAG	63	122
	R	GAAACAGTGCCTTCTGCCAA			R	GAAACAGTGCCTTCTGCCAA		
4749, J2	F	AACCATTAGCAGATGCCAAAA	61	232	F	GGTACAACTAAAATGCCCTCCA	55	199
	R	TCCTCCCTAATTCACTCAACAAA			R	TCCTCCCTAATTCACTCAACAAA		
4749, J3	F	TGCAGTTTAAAGATGATGCAGA	55	249	F	ATGCGGGTGGTCAGGTAATA	58	195
	R	CGCCATTGATGAGAAAGTCC			R	CGCCATTGATGAGAAAGTCC		
4977, J1	F	TCTCTTGTCTGGCCTCCCTA	61	216	F	TCTCTTGTCTGGCCTCCCTA	58	157
	R	TGCACAATTGGCAGACACTAA			R	TGCACAATTGGCAGACACTAA		
4977, J2	F	AAACACATGCGCCTAGAATG	58	247	F	GGTGCCAACACTGGCTGTAT	63	175
	R	TGGTGCGTTAAACACTCTCG			R	TGGTGCGTTAAACACTCTCG		
4978	F	GGGGAACCACTGACCTAACA	63	232	F	GGGGAACCACTGACCTAACA	66	174
	R	ATGGTACAATGGGCCTACGA			R	ATGCACAATTGGCAGACACT		
4995	F	GCGCCTGTAGTCTCAGC	55	186	F	ATGTCCCATGCAATATTTGG	53	116
	R	CCAGCAAAGGATAATAGATGG			R	CCAGCAAAGGATAATAGATGG		
5189, J1	F	GATTTTTGCAAGGCATACCTAA	58	177	F	GATTTTTGCAAGGCATACCTAA	58	154
	R	CATGTTAGCCAGGATGGTC			R	TCTCCTCACCTCGTGATCTG		
5189, J2	F	CAGATTCTAGGTGGCCTTATTTACA	61	290	F	TTTACATAATAGATTGGTGGTGTTT	58	271
	R	CTTGAAGAGGTCCTTCACATCC			R	CTTGAAGAGGTCCTTCACATCC		
5189, J3	F	GTGGAAGTGCAGTTTGATGG	61	215	F	AGTAACTGTGGTAGAGGGTCAAGT	63	171
	R	GCAAGAAACACCTTGTCCTCA			R	GCAAGAAACACCTTGTCCTCA		
5234	F	GCTGAGGCAGGAGAATTGC	53	188	F	GAGGCAGAGGTTGTAGTGAGC	58	126
	R	CATAGATGACAATTTAAGAAATGC			R	AAATTTAGTTTCTATGGATGTAAAGC		
5254	F	GCTCAGGGAAACTCTCTGGA	55	265	F	GCTCAGGGAAACTCTCTGGA	58	233

	**R**	TGTCCTCCAATACTACTAACCACAA			**R**	TCCAGCAAAGGATAATAGATGG		
5613	**F**	TTGACATTGAGGATGTCATTTTG	58	294	**F**	TTGACATTGAGGATGTCATTTTG	55	248
	**R**	CAATGGCTGATCCAGAAGGT			**R**	CAATGGCTGATCCAGAAGGT		

Table S4. Internal oligonucleotides for hybridization

Junction	Internal Oligonucleotide (5′→3′)	T_M (°C)
841, J1	CACTTAAACCCAGGAGGCAG	59,4
841, J2	TGCAGCATCAAAAGAAGGTG	55,3
841, J3	GTACCAGGTTTTGCTTTCGG	57,3
1907	GCTCATCGAAGATTTGAGGG	57,3
2349	AGGCCCGGATAGAAGAAAAA	55,3
3986	GAGCAGGATCCCTGAAGAAG	59,4
4338	TGTTGTTTTGCTTCCTGTGC	55,3
4601	CGAGAACGAAAATGACAGTGA	55,9
4978	TGAGCTGAAACCAAAGTCATT	58
4995	GGAGGATCACCTGAGGTCAG	61,4
5189, J1	TTTCTGCAAGGGTGGAAAAC	55,3
5189, J2	CCAACTTGCAATGGATGTGA	55,3
5189, J3	TGAGACACTGAGGACAAGGTG	59,8
5240	GAGATCGCACCACTGCACTA	59,4
